# The characteristics of the complete chloroplast genome for *Eucalyptus robusta* (Myrtaceae)

**DOI:** 10.1080/23802359.2021.2005491

**Published:** 2021-11-29

**Authors:** Yisen Gao, Jiayi Zhang, Haining Tang, Nuoyi Liu, Guiying Li, Daran Yue

**Affiliations:** a College of Computer Science and Technology, Changchun University of Science and Technology, Changchun, China; b College of Forestry, Hainan University, Haikou, China; c Key Laboratory of Genetics and Germplasm Innovation of Tropical Special Forest Trees and Ornamental Plants, Engineering Research Center of Rare and Precious Tree Species in Hainan Province, College of Forestry, Hainan University, Haikou, China; d Rubber Research Institute, Chinese Academy of Tropical Agricultural Science, Danzhou, China

**Keywords:** *Eucalyptus robusta*, plastome, phylogeny, genome structure, Myrtoideae

## Abstract

*Eucalyptus robusta* is a great tree of Myrtaceae. It is mainly distributed in Southeast provinces of China and Southeast Asian countries. There is no study on the genome of *E. robusta* far. Here we report the complete chloroplast genome of *E. robusta*, assembled from whole-genome high-throughput sequencing data, as a resource for future studies on the taxonomy and evolution of *E. robusta*. The complete chloroplast genome of *E. robusta* is 160,201 bp in length with a typical quadripartite structure, consisting of a large single-copy region (LSC, 88,905 bp), a single-copy region (SSC, 18,498 bp) and a pair of inverted repeats (IRs, 26,399 bp). It was predicted to contain a total of 128 genes, with an overall GC content of 36.86%. Phylogenetic analysis placed *E. robusta* closest to *Eucalyptus globulus.*

*Eucalyptus robusta* is native to Australia, Indonesia, the Philippines and other countries. By 2019, the total area of *E. robusta* plantations in China has reached 580 hm^2^, mainly distributed in Guangdong, Hainan, Yunnan and other regions of China (Tang and Chen 2020). *Eucalyptus robusta* has become a strategic species of fast-growing and high-yield forest in south China. However, a recent study on populations of both varieties using nuclear microsatellite markers found low genetic differentiation between them, suggesting that the current taxonomic treatment may not hold (Song et al. 2016). Also, to our knowledge, there have been no studies on the genome of *E. robusta* up to now. To provide a rich genetic information and improve *E. robusta* molecular breeding in the future, we report and characterize the complete plastid genome sequence of *E. robusta* (GenBank accession number: MZ670598).

The mature and healthy leaves of a single individual of *E. robusta* was sampled from Geze village in Lingshui county, Hainan province (18.63667°N, 109.97111°E). The voucher specimen was deposited in the Herbarium of Guangxi Institute of Botany (accession number: IBK00397639; e-mail address: karst@gxib.cn). The total genomic DNA was extracted from silica gel dried leaves using a modified CTAB method (Doyle and Doyle 1987) and sequenced based on the Illumina pair-end technology. Approximately 11.68 Gb of paired-end (150 bp) sequence data, deposited in SRA database with Accession number SRR15315685 under the Bioproject NO. PRJNA751248 and Biosample NO. SAMN20513345, was used in GetOrganelle (Jin et al. 2020) software to assemble the chloroplast genome. Annotation of the chloroplast genome was performed using the cpgavas and Geseq (Tillich et al. 2017), then manually verified and corrected by comparison with the annotation of *Eucalyptus globulus* (AY780259.1) as the reference.

The complete chloroplast genome sequence of *E. robusta* obtained in this study was 16,201 bp in length, with a small single-copy (SSC) region of 18,498 bp, a large single-copy (LSC) region of 88,905 bp, separated by two inverted repeat (IR) regions of 26,399 bp each. The overall G/C content in the plastome of *E. robusta* is 36.86%. It was predicted to contain 128 genes, including eight rRNA genes, 37 tRNA genes, and 83 proteincoding genes.

To investigate the relationship between Eucalyptus and other genera within the family Myrtoideae, a phylogenetic tree was constructed. We used RAxML (Stamatakis 2006) with 1000 bootstraps under the GTRGAMMAI substitution model to reconstruct a maximum likelihood (ML) phylogeny of 20 published complete plastomes of Myrtoideae, using *Osbeckia stellate* (GenBank accession NC_046486) and *Heterocentron elegans* (GenBank accession NC_051000) (Melastomataceae) as outgroups. According to the phylogenetic topologies, *E. robusta* was closely related to *E. globulus*(GenBank accession AY780259). Most nodes in the plastome ML trees were strongly supported (Figure 1). The complete plastome sequence of *E. robusta* will provides a useful resource for the conservation genetics of this species as well as for the phylogenetic studies for Myrtoideae.

**Figure 1.**
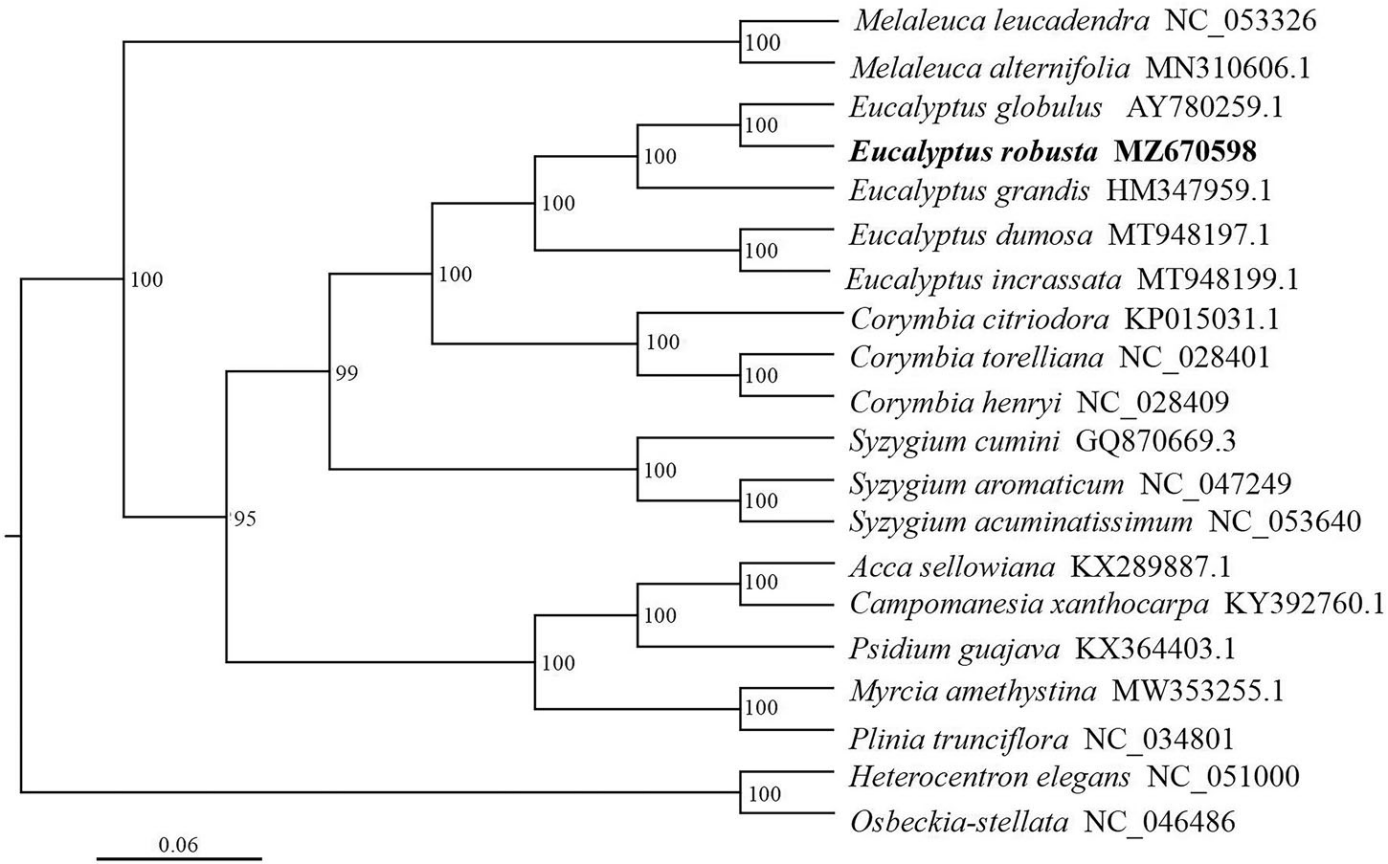
Maximum-likelihood phylogenetic tree based on 20 complete chloroplast genomes. The number on each node indicates the bootstrap value.

## Data Availability

The genome sequence data that support the findings of this study are openly available in GenBank of NCBI at https://www.ncbi.nlm.nih.gov/ with the accession number is MZ670598. Raw sequencing reads used in this study have been deposited in the SRA database of NCBI under accession number SRR15315685. The associated ‘BioProject,’ and ‘Bio-Sample’ numbers are PRJNA751248, and SAMN20513345 respectively.
